# Resveratrol ameliorates neuronal apoptosis and cognitive impairment by activating the SIRT1/RhoA pathway in rats after anesthesia with sevoflurane

**DOI:** 10.17305/bjbms.2021.5997

**Published:** 2021-07-06

**Authors:** Qiaoyun Zhou, Yingfeng Deng, Xuelian Hu, Yinye Xu

**Affiliations:** 1 Department of Anesthesiology, Ningbo Eye Hospital, Ningbo, Zhejiang, China; 2 Department of Anesthesiology, Hwa Mei Hospital, University of Chinese Academy of Sciences, Ningbo, Zhejiang, China

**Keywords:** Resveratrol, neuronal apoptosis, sevoflurane anesthesia, silent information regulator 1/RhoA pathway, post-operative cognitive dysfunction

## Abstract

Studies have shown that long-term exposure to sevoflurane (SEV) may cause post-operative cognitive dysfunction. This study aimed to investigate the effects of resveratrol (RES) treatment on the changes in the cognitive function of rats after prolonged anesthesia with SEV. Seventy-six adult male rats were used in this study. The SEV model was established under continuous anesthesia for 6 hours. Rats were randomly classified into four groups as follows: Control, SEV+vehicle, SEV+pre-RES (RES was administered 24 hours before establishing the SEV model), and SEV+post-RES (RES was administered 1 hour after establishing the SEV model) groups. Neurobehavioral outcomes and the potential mechanism underlying RES-mediated neuroprotection through the silent information regulator 1 (SIRT1)/RhoA signaling pathway were evaluated. The water maze test showed that long-term exposure to SEV may lead to loss of learning and memory ability in rats (*p* < 0.05). Compared with the SEV+vehicle group, the RES treatment groups showed significantly improved neurobehavioral scores (*p* < 0.05). In addition, the SEV+pre-RES group had a better outcome than the SEV+vehicle group on days 1 or 2 (*p* < 0.05), unlike the SEV+post-RES group (*p* > 0.05). Western blotting showed that SIRT1, RhoA, and cleaved Caspase-3 (CC3) expression significantly increased in the SEV+vehicle group (*p* < 0.05), while Bcl2 expression decreased (*p* < 0.05). RES treatment further upregulated SIRT1 and Bcl2 expression and downregulated the expression of RhoA and CC3 (*p* < 0.05). In conclusion, RES treatment improved cognitive dysfunction by reducing neuronal apoptosis in adult rats exposed to SEV. RES partly exerted a neuroprotective effect through the activation of the SIRT1/RhoA signaling pathway.

## INTRODUCTION

Post-operative cognitive dysfunction (POCD) is a common complication of surgery requiring anesthesia. The main clinical manifestations are changes in cognitive function, such as decreased learning and memory abilities, inability to concentrate, and severe personality changes [1]. The current clinical treatment of POCD majorly involves controlling nutrition and fluid intake, maintaining electrolyte balance, and strengthening psychological support, and some patients are administered neurotrophic drugs for treatment [2]. Sevoflurane (SEV) is a widely used clinical inhalation anesthetic. In recent years, an increasing number of researchers have begun to pay attention to the effect of SEV on brain function, especially learning and memory function [3]. Studies have shown that repeated inhalation of SEV can induce pathological changes, such as Aβ deposition and fibrin tangles in the hippocampus [4], leading to the occurrence of POCD [5]. Animal studies have found that exposure of rats to SEV during pregnancy can lead to impaired learning and memory function in their offspring [6], and this effect may be related to SEVs concentration and time of inhalation [7]. In rats, prolonged exposure to high concentrations of SEV can alter the metabolic processes of glucose and amino acid in the brain [8], promote neuroinflammation [9], and affect the brain’s synaptic development [10]. Therefore, the prevention or alleviation of post-operative memory dysfunction caused by SEV has become a key concern for clinicians and researchers.

Resveratrol (RES) is a non-flavonoid polyphenol compound extracted from peanuts, grapes, and blueberries [11]. RES can enter the central nervous system by crossing the blood–brain barrier and has various biological effects, including anti-inflammatory, antioxidant, and anti-apoptotic effects [12]. Studies have shown that RES is a natural plant silent information regulator 1 (SIRT1) agonist, which plays a protective role in the brain by activating SIRT1-related pathways in various diseases [12,13]. Similarly, RES can regulate neuronal growth and differentiation through SIRT1 and prevent neuronal apoptosis by inhibiting p53 activity in Alzheimer’s disease [14]. RES can elicit neuroprotective effects in rats with stroke by modulating the cAMP/AMPK/SIRT1 pathway [15]. RES may regulate inflammation through SIRT1, which has positive effects on aging and age-related diseases [16].

A previous study showed that RES could improve SEV-induced cognitive impairment in newborn mice [17]; however, this phenomenon has not been studied in adult animal models. Therefore, the purpose of this study was to investigate the role of SIRT1/RhoA signaling in the neurotoxicity induced by long-term SEV exposure and the effect and mechanism of RES administered in advance and postoperatively on cognitive impairment in adult rats.

## MATERIALS AND METHODS

### Animals

Seventy-six adult male Sprague–Dawley rats weighing 280-320 g (8-10 weeks old) were used in this study. The experimental protocol was approved by the Animal Care and Use Committee of Ningbo University. The rats were housed in temperature- and humidity-controlled animal quarters with a 12/12 hours light/dark cycle.

### SEV model

The SEV model was established by continuous anesthesia for 6 hours, as previously described [18]. Briefly, rats were placed in plastic boxes supplied with 3% SEV and 40% oxygen (2 L/minutes) for 6 hours. During the modeling process, the temperature of the anesthesia box was maintained at 27°C. The color of the rats’ nose, lips, limbs, and tail was monitored at 30 minutes intervals, and it was confirmed that the rat’s breathing rate remained normal. The rats were modeled and returned to the breeding cage after resuscitation.

### Drug administration

RES was dissolved in dimethyl sulfoxide (DMSO) and diluted with saline. A total volume of 1 mL of the vehicle (DMSO) or RES at three different doses (33, 100, and 300 mg/kg) were administered intraperitoneally (IP) to rats 24 hours before or 1 hour after the establishment of the SEV model.

### Experimental group

**FIGURE 1:**
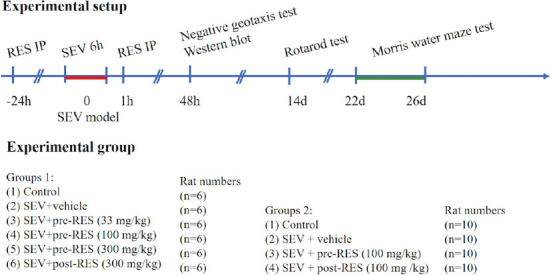
Experimental design and animal groups.

The experimental design and animal groups are shown in Figure 1. To evaluate the effect of RES on short-term neurological outcomes after establishing the model, 36 rats were randomly assigned into six groups (n = 6, per group) as follows: The control group comprised rats unexposed to SEV without RES treatment; the SEV+vehicle group comprised SEV anesthetized rats without RES treatment; the SEV+pre-RES (33 mg/kg), SEV+pre-RES (100 mg/kg), and SEV+pre-RES (300 mg/kg) groups consisted of SEV anesthetized rats, IP administered RES (33, 100, and 300 mg/kg, respectively) 24 hours before the establishment of the model; and the SEV+post-RES (100 mg/kg) group comprised SEV anesthetized rats, IP administered RES 1 hour after the establishment of the model.

To evaluate the effect of RES on long-term neurological outcomes after establishing the model, 40 rats were randomly assigned into the following four groups (n = 10 per group): The control, SEV+vehicle, SEV+pre-RES (100 mg/kg), and SEV+post-RES (100 mg/kg) groups.

### Negative geotaxis test

A negative geotaxis test was performed on day 2 after establishing the SEV model to evaluate short-term neurological function. The rat was placed head down on a wooden board with a 40° incline, and the time taken for the rat to twist its body (torsion angle> 90°) and face head-up was recorded. The reflection test recording was completed within 20 seconds; if the reflection test could not be completed, the recording time was 20 seconds.

### Rotarod test

The rotarod test was performed 2 weeks after establishing the SEV model to evaluate the rats’ long-term neurological function as previously described [19]. The rotation speed started at 5 rpm with an acceleration of 0.4 rpm/seconds, and the time spent by the rat on the roller was recorded.

### Morris water maze

The Morris water maze test was performed to evaluate learning and memory function 22 days after establishing the SEV model. The rats were put into a pool from different directions, and they sought a platform within 1 minute. Rats were trained for 5 days to find the platform, with four trials conducted daily. On day 6, the platform was removed, and the rats’ trajectory and stay time in the target quadrant were recorded. The water maze was divided into four quadrants. After training, the duration for which the rats stayed in the target quadrant was separately recorded using an automated video tracking system (AVTAS v3.3; AniLab, Ningbo, China), which was used to evaluate the changes in the rats’ spatial learning and memory ability.

### Western blotting

Rats were decapitated after performing the short-term neurological function test on day 2. The hippocampal brain tissue was quickly removed and homogenized using cold RIPA lysis buffer (Beyotime, Beijing, China), 1 mM protease inhibitors (Promoter, Wuhan, China), and phosphatase inhibitors (Boster, Wuhan, China). The lysates were centrifuged at 14,000 rpm at 4°C for 30 minutes. The protein concentration was determined using a BCA assay kit (Boster, Wuhan, China). Protein samples (40 mg) were separated by sodium dodecyl sulfate-polyacrylamide gel (10%-15%) electrophoresis and subsequently transferred onto nitrocellulose membranes. The membranes were incubated overnight at 4°C with the following primary antibodies: Anti-SIRT1 (1:1000, sc-74465, Santa Cruz Biotechnology, TX, USA), anti-Bcl2 (1:1000, ab59348, Abcam, USA), anti-Bax (1:1000, ab182734, Abcam, USA), anti-cleaved Caspase-3 (CC3, 1:500, #9661, Cell Signaling Technology, USA), anti-β-actin (1:3000, sc-47778, Santa Cruz Biotechnology, TX, USA), and anti-RhoA (1:1000, sc-418, Santa Cruz Biotechnology, TX, USA). The secondary antibodies (1:3000, Santa Cruz Biotechnology, TX, USA) were incubated at room temperature for 1 hour. Immunoblots were visualized using a chemiluminescence system (ChemiDoc, Bio-Rad, USA) and quantified using the ImageJ software (ImageJ 1.5, NIH, USA).

### Statistical analysis

A one-way analysis of variance (ANOVA), followed by Tukey’s *post hoc* test, was used to compare the four groups. A two-way ANOVA was used to analyze the rotarod and the Morris water maze tests. Statistical analyses and figures were analyzed using GraphPad Prism (GraphPad Software, San Diego, CA, USA), and *p* < 0.05 was considered statistically significant.

## RESULTS

**FIGURE 2:**
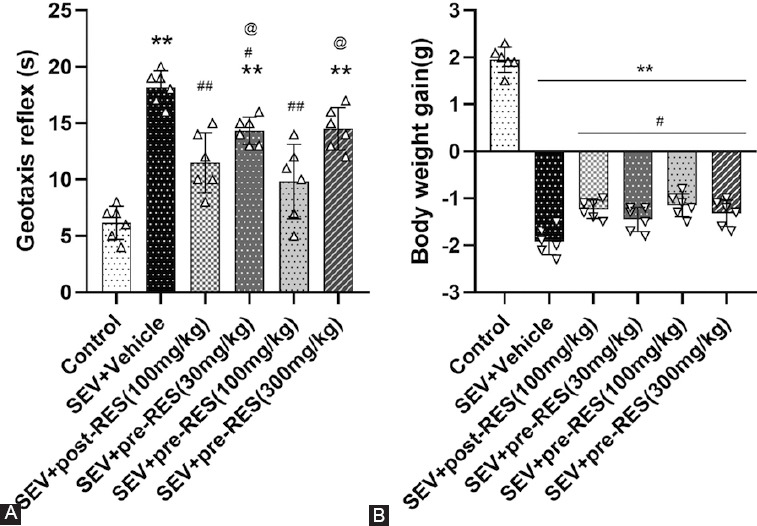
Effect of resveratrol (RES) treatment on short-term neurobehavioral outcomes after establishing the sevoflurane (SEV) model. (A) RES treatment improved short-term neurobehavioral outcomes 24 hours after establishing the SEV model (n = 6 per group). (B) RES treatment can effectively alleviate rats’ weight loss after establishing the SEV model. Data are shown as the mean ± standard deviation (n = 6 per group; one-way repeated measures ANOVA, Tukey’s *post hoc* test). **p < 0.01 versus control group; #*p <* 0.05; ##*p <* 0.01 versus SEV + vehicle group; @ *p <* 0.05 versus SEV + pre-RES group (100 mg/kg).

Seventy-six rats were used in this study, and no mortality was observed in any group. To determine the best RES dosage, three doses, including low (33 mg/kg), medium (100 mg/kg), and high (300 mg/kg) doses, were IP administered to the rats 24 hours before or 1 hour after establishing the SEV model. The negative geotaxis test showed that reflex time significantly increased after administering SEV compared with the control group (*p* < 0.001, Figure 2A). RES treatment decreased the reflex time compared with the SEV+vehicle group (*p* < 0.001), and the medium dose (100 mg/kg) showed the best effect of the three doses administered (*p* < 0.001). Bodyweight measurement results showed that the SEV rat model demonstrated significant weight loss 48 hours after its establishment compared with the control group (*p* < 0.001). The medium (100 mg/kg) and high (300 mg/kg) doses of RES effectively alleviated weight loss in rats (*p* < 0.05, Figure 2B).

**FIGURE 3:**
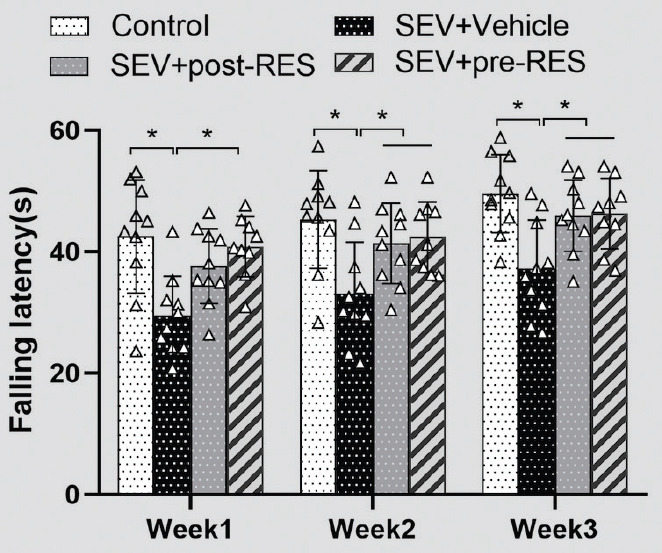
Rotarod test on 3 weekends after establishing the sevoflurane model (n = 6 per group; two-way repeated measures ANOVA, Tukey’s *post hoc* test). *p < 0.05.

The rotarod test demonstrated that the SEV+vehicle group showed a significantly lower latency than the control group (*p* < 0.05) (Figure 3). RES treatment improved rotarod latency 2 and 3 weeks after establishing the SEV model (*p* < 0.05). The SEV+pre-RES group had a better outcome than the SEV+vehicle group at week 1 (*p* < 0.05), unlike the SEV+post-RES group (*p* > 0.05).

**FIGURE 4:**
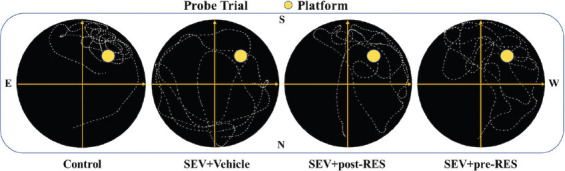
Representative swimming paths of the rats in the probe trial in the four groups. The yellow circles indicate the probe platform.

**FIGURE 5:**
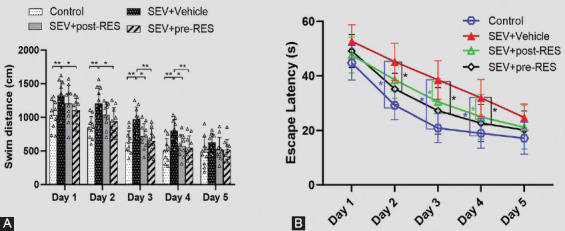
Effects of resveratrol pre-treatment on spatial memory and learning ability after establishing the sevoflurane model. (A) Swimming distance of the Morris water maze test on training days. (B) Escape latency of the Morris water maze test on training days. Data are shown as the mean ± standard deviation (n = 10 per group; two-way repeated measures ANOVA, Tukey’s *post hoc* test). *p < 0.05, **p < 0.01.

**FIGURE 6:**
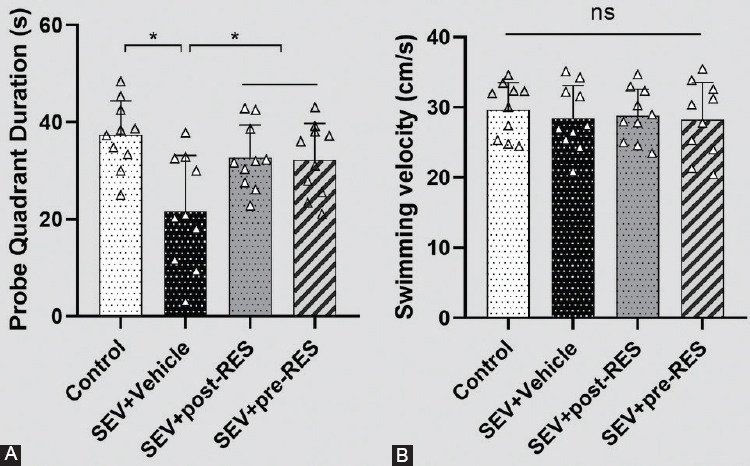
The final test on day 6 of the Morris water maze test. (A) Quantification of the time spent in the probe quadrant in the probe trial on day 6 of the Morris water maze test. (B) Swimming velocities of different groups in the probe trial. Data are shown as the mean ± standard deviation (n = 10 per group, one-way repeated measures ANOVA, Tukey’s *post hoc* test). *p < 0.05; ns: Not significant.

The water maze test showed that SEV administration induced the loss of spatial memory and learning ability in the SEV+vehicle group compared with the control group (Figure 4), resulting in a longer swimming distance to the platform (*p* < 0.01; Figure 5A), more time taken to find the platform (*p* < 0.05; Figure 5B), and less time spent in the target quadrant (*p* < 0.05; Figure 6A). Compared with the SEV+vehicle group, the RES treatment groups showed significantly improved memory and spatial learning deficits, evidenced by more time spent in the probe quadrant (*p* < 0.05; Figure 6A) and shorter swimming distance to the platform on days 3 and 4 (Figure 5A; *p* < 0.05), and less time to find the platform on days 3 and 4 (*p* < 0.05; Figure 5B). In addition, the SEV+pre-RES group had a better outcome than the SEV+vehicle group on days 1 and 2 (*p* < 0.05; Figure 5A-B), unlike the SEV+post-RES group (*p* > 0.05). No significant differences were observed in swimming velocity among the four groups (*p* > 0.05; Figure 6B).

**FIGURE 7:**
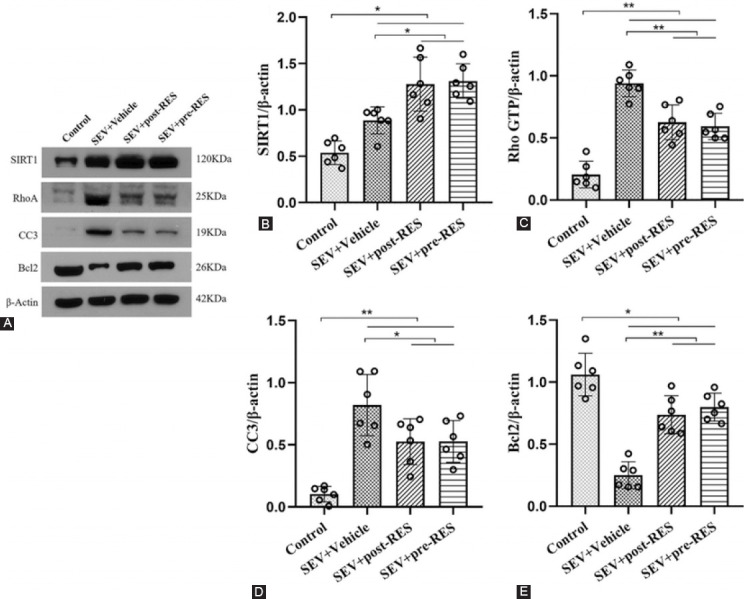
Resveratrol treatment prevented neuronal apoptosis by activating the silent information regulator 1 (SIRT1)/RhoA pathway in the hippocampus of adult rats. (A) Representative Western blotting bands. (B-D) Densitometric quantification of SIRT1, RhoA, cleaved Caspase-3, and Bcl2 based on their ratios to β-actin. Data are shown as the mean ± standard deviation (n = 6, per group one-way repeated measures ANOVA, Tukey’s *post hoc* test). *p < 0.05; **p < 0.01.

Western blotting showed that SIRT1, RhoA, and CC3 expression significantly increased in the SEV+vehicle group (*p* < 0.05; Figure 7A-D), while Bcl2 expression decreased in the SEV+vehicle group compared with the control group (*p* < 0.05; Figure 7E). RES treatment further upregulated SIRT1 and Bcl2 expression and downregulated the expression of RhoA and CC3 in the SEV+post-RES and SEV+pre-RES groups compared with the SEV+vehicle group (*p* < 0.05).

## DISCUSSION

In this study, we demonstrated that RES could significantly prevent SEV-induced brain injury by reducing neuronal apoptosis and cognitive impairment, which was at least partly mediated by the SIRT1/RhoA signaling pathway in adult rats.

A previous study showed that SEV could induce cognitive impairment and degenerative changes in the central nervous system in rats by stimulating the GABA receptor and blocking NMDA receptor-mediated neurotoxic effects [20,21]. Similarly, several animal experiments have shown that SEV exposure can cause neuronal apoptosis (in the hippocampus) and behavioral abnormalities in newborn animals, a phenomenon that is closely related to learning and memory dysfunction [22-24]. In addition, studies have suggested that SEV can cause early POCD after surgery in the elderly [25]. Researchers observed that significant changes in plasma amyloid-beta level [26] and mitophagy impairment [27] in animal models exposed to SEV are related to cognitive dysfunction. Our results suggest that adult rats exposed to SEV for 6 hours required a longer escape latency and path length to locate the platform during the water maze test and spent less time in the target quadrant than those not exposed to SEV. These results suggest that prolonged exposure to SEV impairs spatial learning and memory in adult rats.

RES has been shown to have a broad range of neuroprotective effects in mammals; for example, Huang et al. reported that RES could attenuate β-amyloid-induced neurotoxicity by suppressing inducible nitric oxide synthase production in adult rats [24]. Castro et al. showed that RES also plays an important role in anti-oxidation and anti-inflammation in plasma and brain tissues [28]. Qian et al. suggested that RES reduced brain edema and neuronal apoptosis in a subarachnoid hemorrhage rat model [29]. RES preconditioning can effectively reduce brain damage in neonatal rats after SEV overexposure [17] and alleviate SEV-induced cognitive impairment in elderly rats [30]. Our results suggest that RES treatment can significantly improve spatial memory and learning ability in SEV-induced adult rats. In the early stage of water maze training, the neurologic function of the pre-treated group was better than that of the postoperatively treated group. This observation indicates that pre-treatment with RES is more beneficial for POCD than treatment after anesthesia.

In neurodegenerative diseases, RES was shown to exert a neuroprotective effect through the aggregation of the SIRT1 signaling pathway [31]. RES treatment at different time points could increase neuronal cell activity and inhibit neuronal cell apoptosis *in vitro* [32]. Long-term exposure to SEV causes nerve cell death, characterized by a significant increase in CC3 [33] and RhoA [34] expression in brain tissue. RES treatment has been shown to prevent the activation of Caspase-3 [35] and the RhoA pathway in different diseases [36,37]. Our results showed that SIRT1, CC3, and RhoA expression in the hippocampus was significantly increased, while Bcl2 expression was strongly reduced 48 hours after SEV-induced anesthesia. RES treatment further upregulated SIRT1 and Bcl2 protein levels and downregulated CC3 and RhoA protein levels. These data indicate that SIRT1/RhoA signaling is involved in the beneficial effects of RES treatment after SEV-induced anesthesia.

This study had several limitations. First, RES has also been shown to elicit potent anti-inflammatory and antioxidant effects in neurodegenerative diseases; therefore, we cannot exclude the contribution of these mechanisms to neurological behavior improvements in this study. Second, only the SIRT1/RhoA pathway was evaluated in this study; thus, we cannot exclude the possible contributions of other signaling pathways to the neuroprotective effects of RES treatment. Third, only male rats were evaluated in this study; hence, further related studies involving female rats are warranted. Fourth, we did not verify the blockage of the signaling pathway; thus, this finding needs to be further confirmed in future studies.

## CONCLUSION

RES treatment improved cognitive dysfunction by attenuating neuronal apoptosis in adult rats exposed to SEV for 6 hours. RES exerts a neuroprotective effect, partly through the activation of the SIRT1/RhoA signaling pathway. Pre-treatment with RES was more beneficial for POCD than treatment after anesthesia.
